# Changes in Out-of-Pocket Spending for Common Oral Cancer Medications After the Inflation Reduction Act

**DOI:** 10.1001/jamanetworkopen.2024.32456

**Published:** 2024-09-10

**Authors:** Benjamin Pockros, Chad Ellimoottil, Belal Sbei, Megan Caram, Kristian Stensland

**Affiliations:** 1 Department of Urology, University of Michigan, Ann Arbor; 2 Department of Internal Medicine, University of Michigan, Ann Arbor; 3 School of Medicine, University of Michigan, Ann Arbor

## Abstract

This economic evaluation assesses changes to patient out-of-pocket spending for oral cancer medications before and after the Inflation Reduction Act.

## Introduction

The Inflation Reduction Act (IRA) was enacted in August 2022 and is poised to substantially reduce the costs of prescription drugs in the US. Among its provisions, the law established a new cap on out-of-pocket (OOP) spending on outpatient prescription drugs by Medicare Part D beneficiaries at approximately $3500 in 2024 and $2000 in 2025. Historically there have been no explicit limits on spending, allowing some patients to pay over $10 000 OOP annually on medications.^1^

As policymakers consider strategies to reduce drug spending, including the IRA, understanding the current magnitude of OOP spending on Part D medications is paramount. Evaluating changes to patient spending before and after the law can help quantify and contextualize policy outcomes. This economic evaluation compares out-of-pocket costs for common cancer medications among Part D beneficiaries in 2023 and 2024 to evaluate the financial outcomes of the IRA.

## Methods

We accessed estimated out-of-pocket costs for cancer medications by querying the online Medicare Part D Plan Finder for 2023 and 2024 prices. Drugs included were among the top 10 orally administered brand-name cancer drugs according to Part D spending in 2020.^1^ Part D plans were based on zip code 48103 (Ann Arbor, Michigan) as a representative sample; OOP costs for Part D have been shown to be relatively similar across geographical variation.^2^

Institutional review board approval and informed consent were not required because this study did not involve human participants, in accordance with 45 CFR §46. This study followed the relevant portions of the Consolidated Health Economic Evaluation Reporting Standards (CHEERS) reporting guideline for economic evaluations. A paired *t* test was calculated to compare costs before and after implementation of the IRA. *P* values were 2-sided and statistical significance was set at *P* < .05. Analyses were conducted using SAS version 9.4 (SAS Institute).

## Results

There were a total of 24 Part D plans in 2023 and 20 plans in 2024 available for Medicare beneficiaries in the selected zip code. The mean (SD) OOP cost per year for Part D beneficiaries prescribed oral cancer medications in 2023 was $11 284.03 ($457.80). In 2024, mean (SD) OOP costs per year are $3926.59 ($367.74). The median (IQR) cost savings on annual drug spending from 2023 to 2024 for Part D beneficiaries prescribed oral cancer medications was $7260.12 ($6294.35-$8668.87) per year (Figure and Table). The range of annual OOP savings was from $5694.57 (enzalutamide) to $9872.12 (venetoclax).

**Figure.**
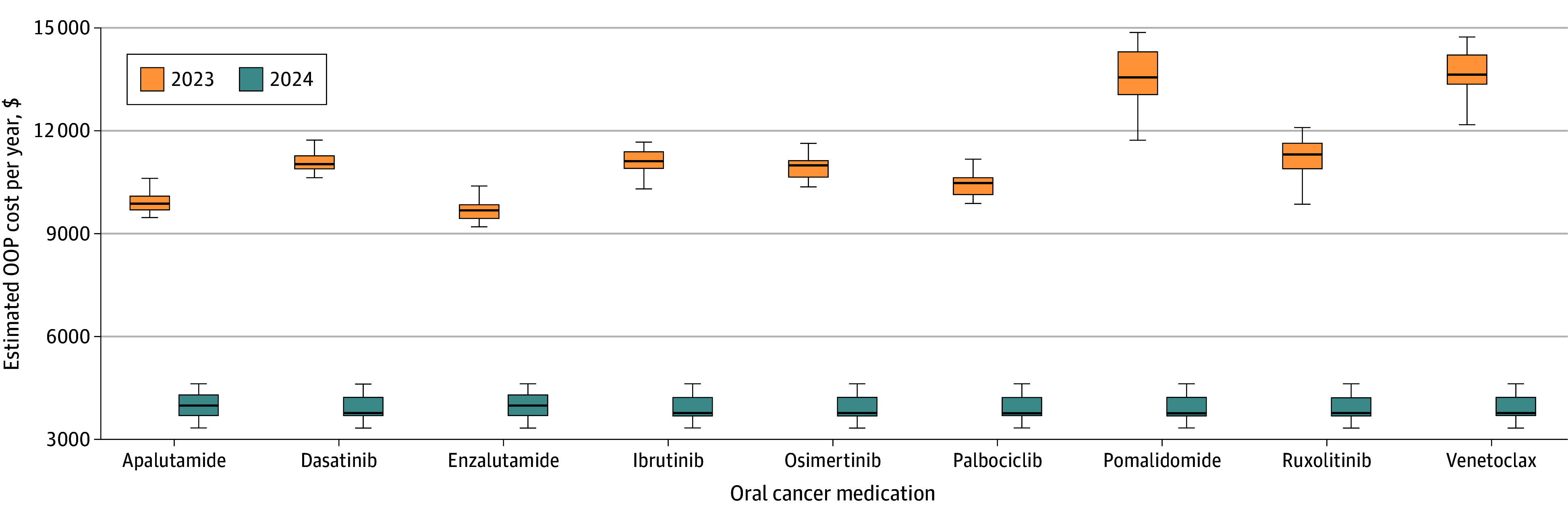
Comparison of Out-of-Pocket (OOP) Costs for Oral Cancer Medications in 2023 and 2024 Drugs included were among the top 10 orally administered brand-name cancer drugs according to Part D spending in 2020. Estimated OOP cost per year is based on Part D plans in zip code 48103. Box plots demonstrate the median annual OOP cost per medication, in addition to IQR and the lowest and highest OOP costs based on all available Part D plans.

**Table.** Mean Out-of-Pocket (OOP) Costs for Oral Cancer Medications in 2023 and 2024

Medication	OOP cost, mean (SD)		*P* value
	2023	2024	
Apalutamide	$9931.98 ($390.40)	$3972.85 ($370.47)	<.001
Dasatinib	$11 042.54 ($344.34)	$3913.37 ($366.96)	<.001
Enzalutamide	$9691.56 ($399.68)	$3972.85($370.47)	<.001
Ibrutinib	$11 066.40 ($404.83)	$3913.37 ($366.96)	<.001
Osimertinib	$10 895.21 ($386.09)	$3913.37 ($366.96)	<.001
Palbociclib	$10 400.50 ($381.42)	$3913.37 ($366.96)	<.001
Pomalidomide	$13 591.88 ($742.36)	$3913.37 ($366.96)	<.001
Ruxolitinib	$11 253.73 ($468.10)	$3913.37 ($366.96)	<.001
Venetoclax	$13 682.48 ($603.00)	$3913.37 ($366.96)	<.001

## Discussion

This economic evaluation found that the OOP cap legislated by the IRA may save patients enrolled in Part D plans a median of $7260 in 2024 for oral cancer medications. These savings will likely continue to grow as the OOP cap decreases from about $3500 in 2024 to $2000 in 2025.

High OOP costs are associated with lower rates of adherence, increased rates of hospitalization, and mortality.^3^ Patients with cancer are particularly vulnerable to financial distress and medical debt.^4^ This study suggests that the IRA may substantially reduce OOP drug spending for patients with cancer enrolled in Medicare Part D Plans. Furthermore, these results suggest that legislative policy can be effective in lowering patient costs. A $2000 cap on OOP drug spending would have yielded savings for 1.5 million patients insured by Medicare Part D in 2021.^5^

This analysis is limited to a single zip code, which may not be precisely representative of other areas. All cost data are estimates from an online database and may differ from actual costs due to supplement coverage or pharmacy distribution. Despite these limitations, we found that patients prescribed brand-name oral cancer medications may save thousands of dollars in annual drug spending due to the legislative effects of the IRA. Understanding this magnitude of cost savings is vital as policymakers consider potentially extending the cap to include patients with commercial insurance.^6^

## Notes

Supplement.
Data Sharing Statement
