# Supplementary material for: Changes in Out-of-Pocket Spending for Common Oral Cancer Medications After the Inflation Reduction Act
Source: JAMA Netw Open. 2024 Sep 10;7(9):e2432456. doi: 10.1001/jamanetworkopen.2024.32456 (PMC11388030; doi:10.1001/jamanetworkopen.2024.32456)
Supplement: Supplement. — Data Sharing Statement [file jamanetwopen-e2432456-s001.pdf]

Data Sharing Statement

Pockros. Changes in Out-of-Pocket Spending for Common Oral Cancer Medications After the Inflation Reduction Act. *JAMA Netw Open*. Published September 10, 2024.
doi:10.1001/jamanetworkopen.2024.32456

Data
Data available: No

Additional Information
Explanation for why data not available: Data is available per request.
